# Hinge-deleted IgG4 blocker therapy for acetylcholine receptor myasthenia gravis in rhesus monkeys

**DOI:** 10.1038/s41598-017-01019-5

**Published:** 2017-04-20

**Authors:** Mario Losen, Aran F. Labrijn, Vivianne H. van Kranen-Mastenbroek, Maarten L. Janmaat, Krista G. Haanstra, Frank J. Beurskens, Tom Vink, Margreet Jonker, Bert A. ‘t Hart, Marina Mané-Damas, Peter C. Molenaar, Pilar Martinez-Martinez, Eline van der Esch, Janine Schuurman, Marc H. de Baets, Paul W. H. I. Parren

**Affiliations:** 1 Department of Psychiatry and Neuropsychology, School for Mental Health and Neuroscience, Maastricht University, Maastricht, The Netherlands; 2 Genmab, Utrecht, The Netherlands; 3 Department of Clinical Neurophysiology, Maastricht University Medical Center, Rijswijk, The Netherlands; 4 Biomedical Primate Research Centre, Rijswijk, The Netherlands; 5 Department of immunohematology and Blood Transfusion, Leiden University Medical Center, Leiden, The Netherlands; 6 University of Groningen, University Medical Center, Department of Neuroscience, Groningen, The Netherlands; 7 Neuroimmunology Group, Biomedical Research Institute (BIOMED), Hasselt University, Diepenbeek, Belgium

## Abstract

Autoantibodies against ion channels are the cause of numerous neurologic autoimmune disorders. Frequently, such pathogenic autoantibodies have a restricted epitope-specificity. In such cases, competing antibody formats devoid of pathogenic effector functions (blocker antibodies) have the potential to treat disease by displacing autoantibodies from their target. Here, we have used a model of the neuromuscular autoimmune disease myasthenia gravis in rhesus monkeys (*Macaca mulatta*) to test the therapeutic potential of a new blocker antibody: MG was induced by passive transfer of pathogenic acetylcholine receptor-specific monoclonal antibody IgG1-637. The effect of the blocker antibody (IgG4Δhinge-637, the hinge-deleted IgG4 version of IgG1-637) was assessed using decrement measurements and single-fiber electromyography. Three daily doses of 1.7 mg/kg IgG1-637 (cumulative dose 5 mg/kg) induced impairment of neuromuscular transmission, as demonstrated by significantly increased jitter, synaptic transmission failures (blockings) and a decrease in the amplitude of the compound muscle action potentials during repeated stimulations (decrement), without showing overt symptoms of muscle weakness. Treatment with three daily doses of 10 mg/kg IgG4Δhinge-637 significantly reduced the IgG1-637-induced increase in jitter, blockings and decrement. Together, these results represent proof-of principle data for therapy of acetylcholine receptor-myasthenia gravis with a monovalent antibody format that blocks binding of pathogenic autoantibodies.

## Introduction

Autoimmune disorders affect more than 5% of the general population, while for reasons that are poorly understood, the incidence of these disorders is currently increasing. In recent years, autoimmune mechanisms have been discovered in many diseases of the nervous system as being responsible for both neurological as well as psychiatric symptoms. Currently available broad-spectrum immunosuppressive drugs are routinely used to treat different autoimmune diseases, but often cause serious side-effects while generally taking four to fifteen months before achieving clinical remission. Antigen-specific therapies offer the possibility to avoid general immunosuppression and its associated risk for infections.

Myasthenia gravis (MG) is one of the best-understood autoimmune disorders and is characterized by muscle weakness as a result of impaired neuromuscular transmission. This is caused by autoantibodies against postsynaptic membrane proteins at the neuromuscular junction (NMJ). In most MG patients (~85%), the auto-antibodies are directed to the muscle nicotinic acetylcholine receptor (AChR)^1^ and induce loss of the AChR by means of complement-mediated lyses of the postsynaptic membrane^2, 3^ and cross-linking-induced degradation of the AChR (antigenic modulation)^4^. The muscle AChR is highly susceptible to antibody cross-linking for two reasons: First, the AChR is densely clustered^5^ and second, each AChR pentamer contains two identical alpha subunits, thus allowing cross-linking of various AChRs by bivalent antibodies against the alpha subunit. More than half of AChR-specific autoantibodies in MG patients is directed to the main immunogenic region (MIR) on the AChR alpha subunits^6^, as exemplified by the human monoclonal antibody IgG1-637^7, 8^. Although the MIR is important for expression and assembly of the AChR, MIR-specific autoantibodies do not interfere with AChR function, such as recognition of acetylcholine released from nerve terminals and opening of the receptor channel^8^. The exposed orientation of the MIRs of adjacent AChRs allows bivalent binding of MIR-antibodies, resulting in rapid AChR degradation. In contrast to the overall AChR antibody titer, the MIR antibody titer is strongly correlated to severity of muscle weakness in MG patients^6^. The pathogenicity and restricted epitope-specificity of AChR-specific autoantibodies makes the MIR an attractive target for blocker therapy with non-pathogenic antibodies^9–11^. Although autoantibodies can bind to various conformational epitopes within the MIR (e.g. 1–32 and 60–81^8^) the binding area typically buried upon antibody binding is a circle with a diameter of 3.1 nm. Therefore, a blocking antibody has the potential to shield a large part of the AChR, which has a diameter of ~6 nm^5^. In passive transfer MG (PTMG) experiments using rats and mice, blocking antibody fragments directed against the MIR have been shown to prevent muscle weakness^10, 12^. *In vitro*, a single chain-Fv antibody fragment of IgG1–637 blocked binding of MG patient autoantibodies by 31.4% (range 2–77.4%)^9^. Similarly, varying degrees of competition of MG patients’ antibodies were observed with Fab-637 (ranging between <10% and 100%)^7^.

In rhesus monkeys, IgG1-637 causes destruction of the postsynaptic membrane, thereby inducing MG symptoms of muscle weakness^11^. In this model, an IgG4 version of the same antibody, IgG4-637, did not induce PTMG, and moreover, prevented IgG1-637-mediated muscle weakness^11^. This protective effect was explained by the inability of IgG4-637 to activate rhesus monkey complement, and the intrinsic ability of human IgG4 molecules to engage in Fab-arm exchange leading to functional monovalency; thus preventing antigenic modulation through cross-linking^11, 13, 14^.

Although these results are promising from a therapeutic point of view, Fab-arm exchange is a slow process *in vivo* ^11, 15^, which implies that therapeutic IgG4 would only gradually become functionally monovalent and thus would initially be able to cause harmful cross-linking-induced antigenic modulation, directly when administered to patients or animals. Moreover, indirect cross-linking through residual interactions of IgG4 with Fcγ receptors^16–18^ may represent an alternative route for antigenic modulation independent of functional monovalency. In addition, during Fab-arm exchange, bispecific antibodies will be generated with unknown partner-specificities, which may result in unpredictable pharmacodynamics. Taken together, these considerations suggest that for immunotherapy of MG an alternative antibody format with superior non-activating (no complement activation or Fcγ-receptor interaction) and non-cross-linking properties, compared to IgG4, is desired for clinical implementation.

In humans, the clinical presentation of AChR-MG may comprise fluctuating fatigability, drooping of eyelids (ptosis), difficulty in swallowing (dysphagia), shortness of breath (dyspnea) and proximal muscle weakness on repetitive use of the muscles^19^. Apart from the analysis of serum autoantibodies against the AChR, several functional tests are used for diagnosis, including injection of a cholinesterase inhibitor (Tensilon test), electromyography (EMG) and single-fiber electromyography (SFEMG). Of these tests, SFEMG is the most sensitive for detecting defects in neuromuscular transmission. In experimental autoimmune MG, rhesus monkeys display the same range of clinical symptoms as patients with MG (e.g. fatigability, hypoactivity, ptosis, dysphagia, anorexia), which can be scored by observing the behavior^20, 21^. Unlike in chronic models, in PTMG these symptoms are reversible and dose-dependent^11^.

In this study we evaluated hinge-deleted IgG4 (IgG4Δhinge) as therapeutic antibody format in PTMG in rhesus monkeys. By employing diagnostic EMG and SFEMG, we could study the therapeutic effects in this animal model and show that IgG4Δhinge protected the NMJ against a myasthenogenic autoantibody.

## Results

### Generation and functional characterization of IgG4Δhinge-637

**Figure 1:**
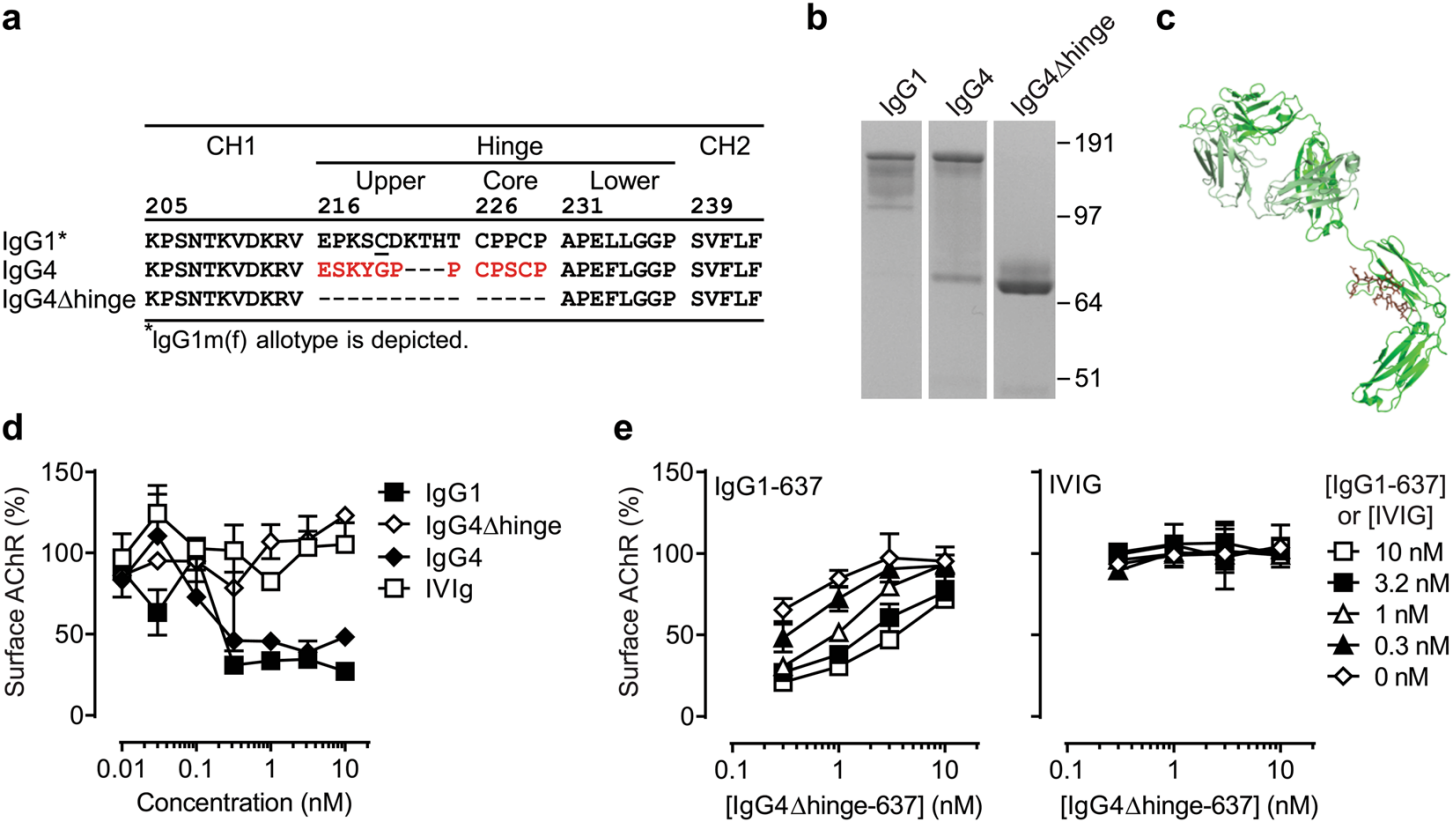
Design and *in vitro* characterization of IgG4Δhinge. (**a**) Sequence alignment of residues 205–234 (EU-numbering convention) of human IgG1, IgG4 and IgG4Δhinge. (**b**) Purified human IgG1, IgG4 and IgG4Δhinge variants of mAb 637 analyzed by non-reducing SDS-PAGE (image is cropped to show relevant bands, the complete gel is shown in supplementary Fig. 5). (**c**) In silico model of IgG4Δhinge based on crystal structures 1ADQ^61^ (CH2-CH3 domains), 1MCO^62^ (Δhinge region) and 1HZH^63^ (Fab region). HCΔhinge depicted in green, LC depicted in pale green. (**d**) AChR surface down-modulation on TE671 cells by purified variants of mAb 637 as indicated. Pooled intravenous immunoglobulin (IVIg) is included as negative control (**e**) Inhibition of (auto-) antibody-mediated AChR surface down-modulation on TE671 cells by IgG4Δhinge-637. Different concentrations of the challenge autoantibody (IgG1-637) or a negative control (IVIG) were tested in combination with IgG4Δhinge-637; each curve corresponds to a single concentration of challenge antibody (0–10 nM) as shown in the key on the right.

In order to increase the non-activating and non-cross-linking properties of human IgG4, constructs lacking the genetic hinge exon were generated, resulting in hinge-deleted IgG4 (IgG4Δhinge) molecules (Fig. 1a). Especially the absence of inter heavy-chain disulfide linkages in IgG4Δhinge, as a result of C226 and C229 deletion, is thought to eliminate residual Fcγ receptor interactions, like observed for IgG1 and IgG3^22, 23^. The hinge deletion in combination with the relatively weak CH3-CH3 interface interaction in human IgG4, minimizes heavy chain dimerization and thus antigen cross-linking^24^.

To study this, an IgG4Δhinge version of mAb 637 was constructed and compared to IgG1-637 and IgG4-637 by non-reducing SDS-PAGE (Fig. 1b). Whereas IgG1-637 remained intact under non-reducing conditions, IgG4-637 displayed a mixture of intact (H2L2) and half-molecules (HL), as is characteristic for monoclonal wild-type human IgG4 molecules^25–27^. As expected, deletion of the genetic hinge region resulted in an exclusive population of non-covalently linked half-molecules (modeled in Fig. 1c).

The inability of IgG4Δhinge to activate complement was confirmed by C1q-binding ELISA (Supplementary Fig. 1a) and complement dependent cytotoxicity (CDC) assay (Supplementary Fig. 1b) using an IgG4Δhinge version of HuMab 7D8^28^, directed against the CD20 antigen and shown to potently induce CDC as IgG1. Furthermore, elimination of residual FcγRI receptor interaction of IgG4Δhinge was confirmed by flow-cytometry (Supplementary Fig. 1c). Together, these data show that IgG4Δhinge has superior non-activating properties compared to IgG4.

Lack of inter heavy-chain disulfide linkage has been shown to influence antibody serum half-life^29^. Furthermore, as half-molecules contain only one instead of two binding sites for the neonatal Fc receptor (FcRn)^30^, their rescue from the IgG degradation pathway is likely to be impaired^31^. To determine the pharmacokinetics of IgG4Δhinge, single doses of IgG4-637 or IgG4Δhinge-637 were injected into Balb/c mice, cynomolgus monkeys (*Macaca fascicularis*) and human FcRn transgenic (Tg) mice^32^ and serum levels were followed over time (Supplementary Fig. 2a–c). As anticipated, IgG4Δhinge-637 was cleared 2.5, 2 and 3.2 times faster compared to IgG4-637 in the respective animal models (Supplementary Fig. 2d). IgG4Δhinge-637, however, was still protected from catabolic degradation as shown by the much faster clearance of the F(ab’)_2_ fragments (Supplementary Fig. 2a,d).

### Protection against auto-antibody-induced AChR down-modulation *in vitro*

Bivalent targeting of the AChR, by either IgG1-637 or IgG4-637, induced AChR surface down-modulation (antigenic modulation) *in vitro* (Fig. 1d), as described^4, 33–35^. In contrast, IgG4Δhinge-637 did not reduce AChR surface expression, showing that IgG4Δhinge-637 is non-cross-linking (Fig. 1d). To investigate whether IgG4Δhinge-637 could protect against AChR surface down-modulation, serial dilutions of IgG4Δhinge-637 were co-incubated with a fixed optimal concentration of IgG1-637. Indeed, IgG4Δhinge-637 could effectively inhibit IgG1-637-mediated loss of AChR expression (Fig. 1e). Pooled intravenous immunoglobulin (IVIg), included as a negative control, did not affect AChR expression by itself, and no significant changes in AChR expression were observed when co-incubated with IgG4Δhinge-637 (Fig. 1e). These data showed that IgG4Δhinge-637 is able to neutralize IgG1-637-mediated down modulation of the AChR *in vitro* and limited the loss of AChR expression.

### Passive transfer MG and clinical evaluation in rhesus monkey model

Since establishment of the PTMG model^11^, improved animal housing conditions had resulted in increased weight, muscle mass and fitness of experimental rhesus monkeys. Therefore, a pilot study with six female rhesus monkeys was initiated in order to check the validity of the experimental conditions for monkeys raised under the new housing conditions. The animals were each challenged intravenously with 1.7 mg/kg/day IgG1-637 on three consecutive days, adding up to a cumulative dose of 5 mg/kg IgG1-637, which had been shown to result in clinical symptoms of MG in a previous study using lighter animals^11^. In the present study, neuromuscular transmission was investigated using single fiber electromyography (SFEMG), to increase sensitivity of the analysis. Furthermore, clinical observation, blood sampling, decrement measurements of the compound muscle action potential (CMAP) and intercostal biopsies were all included in the clinical evaluation.

Intravenous (i.v.) injections with IgG1-637 were well tolerated and no acute adverse effects were observed. Based on clinical observations, none of the animals showed muscle weakness. Baseline (before antibody treatment) mean consecutive difference (MCD) or “jitter” values were recorded in these animals ranging from 9–19 µs (Supplementary Fig. 3), similar to those found in healthy humans (typically between 10 and 20 µs^36, 37^). Seven days after the first injection of the three daily doses of IgG1-637, the jitter values in the six rhesus monkeys increased significantly, with MCD values ranging from 39 to 130 μs (Supplementary Fig. 3).

As a considerable effect on jitter could be measured by SFEMG, without evident muscle weakness, the 5 mg/kg IgG1-637 dose was chosen as a model for subclinical MG. Since jitter values above 91 μs are strongly predictive for respiratory muscle weakness in MG patients^38^, we considered it possible that moderate clinical symptoms were not observed due to the natural behavior of rhesus monkeys to avoid showing weakness in order to preserve social hierarchy^39^. No higher IgG1-637 dose was attempted for our studies to avoid the risk of inducing a myasthenic crisis. All other recorded data were blinded, included in the subsequent study and (re-) analyzed.

### Electrophysiological evaluation of IgG4Δhinge-637 treatment in passive transfer MG

**Figure 2:**
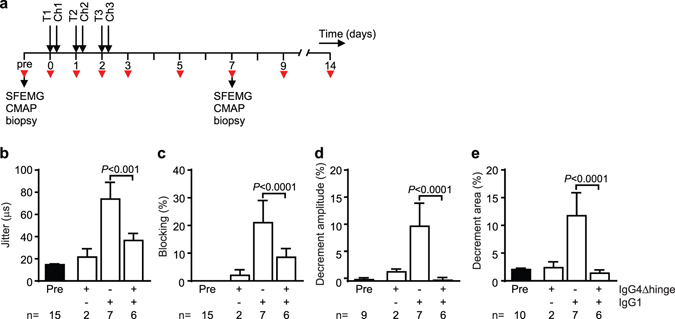
Electrophysiological evaluation of IgG4Δhinge-637 treated rhesus monkeys. (**a**) Treatment schedule. Monkeys were treated with PBS or IgG4Δhinge-637 (IgG4Δhinge) on three consecutive days (T1-T3), 6 hours prior to challenge (Ch1-Ch3) with PBS or IgG1-637 (IgG1). Intercostal muscle biopsies, single fiber electromyography (SFMG) and compound muscle action potential (CMAP) measurements during repetitive nerve stimulation were performed before (pre) and 7 days after start of treatment. Red arrowheads indicate blood sampling. Mean consecutive difference (MCD) of the delay between motor nerve stimulation and muscle fiber action potential (Jitter) (**b**) and neuromuscular transmission failures (Blockings) (**c**) in the orbicularis oculi muscle in different treatment groups. Decrement amplitude (**d**) and area (**e**) in the CMAP of the extensor digitorum brevis muscle in different treatment groups. Reference values were recorded before start of treatment (Pre). Data represent mean ± SEM.

Subclinical MG was induced in seven additional animals to assess the therapeutic effect of IgG4Δhinge-637. For this, the animals received three daily intravenous doses of 10 mg/kg IgG4Δhinge-637 (n = 6) or PBS (n = 1), six hours prior to challenge with IgG1-637. The dosing of IgG4Δhinge-637 was based on the protective dose of IgG4-637 (three daily doses of 5 mg/kg) in a previous study^11^, compensated for the two-fold faster clearance compared to IgG4 as determined in cynomolgus monkeys (Supplementary Fig. 2). Two additional control animals only received IgG4Δhinge-637 and no IgG1-637 challenge. Clinical signs of MG were monitored as described above (Fig. 2a).

Blinded (re-)analysis of the SFEMG data of the untreated rhesus monkeys (seven animals total) yielded control values for jitter in rhesus monkeys. The mean MCD obtained was 14.83 ± 3.39 μs (range 9–19 μs; Fig. 2B) with an upper limit (mean ± 3 SD) of 24.99 μs. The mean control MCD for all individual potentials (n = 352) obtained was 14.88 ± 6.45 μs (range 5–55 μs) with an upper limit of 34.23 μs. Administration of IgG4Δhinge-637 alone did not significantly increase the jitter (Fig. 2b). Challenge with IgG1–637 significantly (P < 0.001) increased the jitter compared to controls (Fig. 2b). Animals receiving IgG4Δhinge-637 treatment prior to pathogenic challenge with IgG1-637 showed significantly (P < 0.001) reduced jitter compared to the group challenged with IgG1-637 alone (Fig. 2b).

Subsequently, we analyzed the frequency of neuromuscular blockings, the failure to elicit an action potential in the muscle fiber upon stimulation of the motor nerve (Fig. 2c). No blocking events were observed in the animals before antibody administration. A few events (4%) of blockings were observed in one out of two animals injected with the IgG4Δhinge-637 alone, indicating a possible minor effect on synaptic transmission. In six out of seven animals challenged with IgG1-637, blocking events were observed with an average blocking rate per animal of 24.1% (Fig. 2c). Similarly, blocking was observed in five out of six rhesus monkeys treated with IgG4Δhinge-637 six hours prior to injection with IgG1-637. In these animals, the number of blockings induced by IgG1-637 was significantly (P < 0.0001) reduced to 8.6% by treatment with IgG4Δhinge-637 (Fig. 2c).

During repetitive nerve stimulation, the amount of neurotransmitter that is released at each subsequent nerve impulse decreases, especially at higher stimulation frequencies. In combination with low levels of AChRs, this leads to increasing numbers of neuromuscular blocking events during repetitive nerve stimulation. As a result, the CMAP decreases during repetitive stimulations of the muscles, which is referred to as a decrement of the CMAP response. In clinical practice a decrement of 10% or more at a stimulation frequency of 3 Hz (including both the amplitude and the area under the curve) is considered confirmation of the diagnosis of MG in individual patients^40^. More recently, a 7–8% cutoff was found to increase sensitivity of this test for MG diagnosis by 6–11%, while preserving high specificity of >95%^41^. CMAPs were recorded from the extensor digitorum brevis (EDB) muscle of the foot during repeated stimulation of the peroneal nerve below the fibular head. Four out of seven animals had 7% decrement or more in the untreated IgG1-637-challenged group (see Supplemental Table 2). None of the animals in the other experimental groups reached these levels. There were significant differences of the average decrement values between the groups (Fig. 2d and e) that paralleled the aforementioned changes in jitter values (Fig. 2b). Amplitude and area decrement values measured in animals in the untreated and in the IgG4Δhinge-637 control groups ranged between −3% and +5%. Administration of IgG1-637 significantly increased average decrement values to 11% for amplitude and 13% for area decrement. Treatment with IgG4Δhinge-637 significantly reduced these IgG-637-induced decrement values to −0.3% and 1.4%, respectively (both P < 0.0001) (Fig. 2d and e).

### Electron microscopic analysis of intercostal neuromuscular junctions

**Figure 3:**
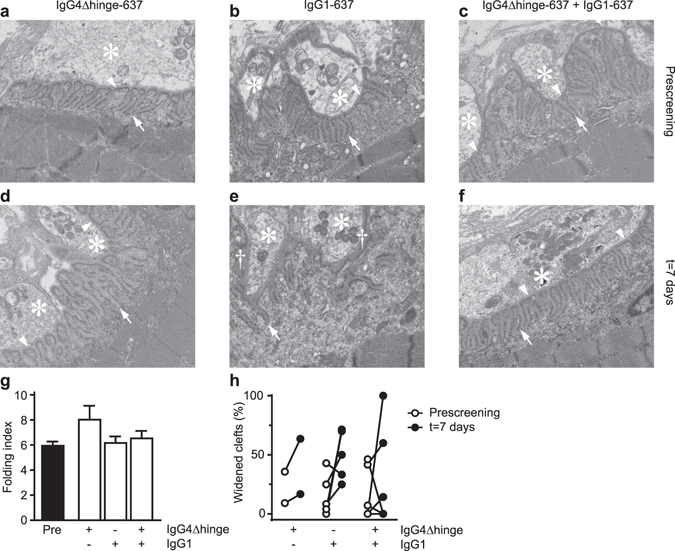
Electron microscopic analyses of intercostal neuromuscular junctions in rhesus monkeys. Transmission electron micrographs showing representative nerve boutons of 3 animals, either before (‘Prescreening’, **a**,**b**,**c**) or seven days after challenge with IgG4Δhinge-637 (**d**), IgG1-637 (**e**) or the combination of IgG4Δhinge-637 and IgG1-637 (**f**). Each micrograph has the dimension of 5 × 6 µm. Asterisks indicate nerve terminals/boutons and arrowheads point at the (intact) primary synaptic clefts. Arrows point to normal secondary postsynaptic clefts/folds; the daggers in panel e indicate widening of the primary synaptic cleft, where the presynaptic and the postsynaptic membrane were separated from each other. (**g**) The folding index (length of postsynaptic membrane/length of the corresponding presynaptic membrane), a measure of the degree of postsynaptic folding. (**h**) Blinded scoring of the normal versus widened synaptic clefts.

Electron micrographs were used for qualitative and quantitative evaluation of the degree of postsynaptic damage at synaptic boutons in intercostal muscle biopsies taken before, and one week after the first antibody administration (Fig. 3a–f). In line with the mild clinical symptoms, no significant differences of the folding index (a measure for folding complexity of the postsynaptic membrane) were observed between the experimental groups (Fig. 3g). Nonetheless, a widening of the primary and secondary synaptic cleft (indicating ultrastructural damage of the NMJ) was frequently observed (see Fig. 3e). Because no quantitative morphometric analysis is currently available for this change, all images were subjected to blind qualitative scoring (Fig. 3h). This indicated a significant widening of the primary synaptic cleft in the IgG1-637-challenged muscles as compared to the untreated control muscles. In contrast, significant cleft widening was not observed in combination with the IgG4Δhinge-637 treatment (Fig. 3h).

### Plasma and NMJ levels of active complement

**Figure 4:**
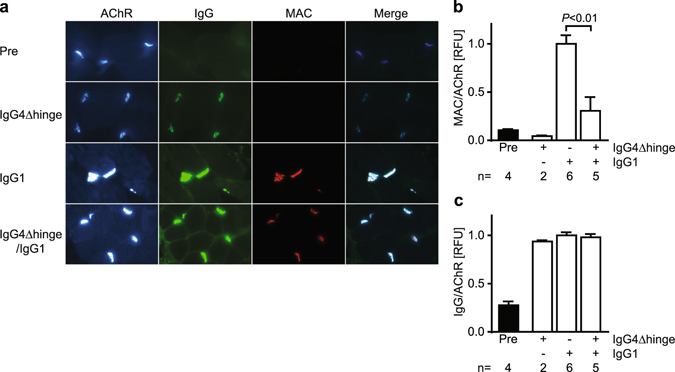
Quantitative Immunofluorescent analysis of NMJ endplates in rhesus monkey intercostal biopsies. Biopsies were obtained from each animal before (“Pre”) or 7 days after antibody challenge with IgG4Δhinge-637, IgG1-637 or the combination of both. (**a**) Representative photomicrographs from the four groups showing staining of the AChR (detected by alpha-bungarotoxin fluorescence), IgG (IgG + IgG4Δhinge), the membrane attack complex (MAC) and a merged image to show colocalization. Relative fluorescence intensities (RFU) of (**b**) MAC staining and (**c**) human IgG staining was normalized with AChR expression in individual endplates. Intensities were quantitated in a total of 589 endplates (5-158 endplates per biopsy) and averaged per biopsy. The number n indicates the number of animals/biopsies analyzed for each condition. Data represent means ± SEM.

In addition to antigenic modulation, the pathogenicity of IgG1-637 may be attributed to antibody-mediated complement activation. To investigate the role of complement in the PTMG model in rhesus monkey, levels of C4b/c, as a measure for overall complement activation, were quantified at different time-points in the plasma of all monkeys. No significant changes in C4b/c levels were observed in time or between groups (Supplementary Fig. 4). Moreover, we analyzed formation of membrane attack complex (MAC, C5b-9) in intercostal muscle biopsies taken before and after antibody treatment (Fig. 4). While little or no MAC staining was found in endplates of unchallenged and IgG4Δhinge-637-treated monkeys, a consistently very strong MAC staining colocalized with human IgG and alpha-bungarotoxin staining in IgG1-637-treated monkeys (Fig. 4a). Treatment with IgG4Δhinge-637 prevented MAC deposition in endplates of IgG1-637 challenged animals by 70% (P < 0.01) (Fig. 4b). Since total human IgG levels (IgG1 + IgG4Δhinge) were comparable between these groups (Fig. 4c), these data suggest that IgG4Δhinge partially blocked binding of IgG1 at the intercostal NMJs.

## Discussion

The presence of circulating autoantibodies is a hallmark of autoimmune disease (AID). In some organ-specific AIDs, like MG, the autoantibodies are solely responsible for the elicitation of pathogenic effects and frequently display restricted epitope-specificity^7, 42, 43^. In such cases, displacing pathogenic autoantibodies from their target with blocker antibodies (competing antibody formats devoid of pathogenic effector functions) represents a potential treatment strategy. The validity of this concept has been demonstrated previously with MIR-specific antibody formats in AChR MG^7, 11^ and an aquaporin-4 (AQP4)-specific antibody format in neuromyelitis optica^44^.

In MG, the autoantibody-mediated pathogenic effector functions include antigenic modulation through auto-antibody-mediated receptor cross-linking^4^ and complement-mediated lysis of the post-synaptic membrane^2, 3^. The therapeutic potential of Fab fragments and post-exchange IgG4 has been shown before^7, 11^, as both can displace pathogenic serum auto-antibodies without being able to activate complement or induce receptor cross-linking, due to their (functional) monovalency. However, the rapid clearance of Fab fragments from the circulation and the potential of IgG4 for indirect cross-linking via residual Fc-receptor interactions are clear disadvantages of these approaches^16–18^. In addition, IgG4 only gradually becomes functionally monovalent through Fab-arm exchange upon administration in patients and thus could be pathogenic by inducing cross-linking through bivalent interaction at the initiation of therapy^11, 15^. The present study describes the use of IgG4Δhinge as format for blocker therapy of MG in a non-human primate model. The patient-derived IgG4Δhinge blocked cell surface AChR binding of a pathogenic IgG1 and prevented antigenic modulation *in vitro*. Treatment of pathogenic IgG1-challenged rhesus monkeys with IgG4Δhinge significantly improved the neuromuscular transmission defects *in vivo* as measured by electromyography and microscopic analysis of muscle biopsies. The relatively long half-life in combination with monovalent interaction makes this non-pathogenic antibody format superior to Fab fragments^7^, post-exchange IgG4^11^ or silenced IgG1 formats^44^ and suitable for clinical applications in MG.

Without covalent disulfide linkages in the hinge region, human IgG4 half-molecules are non-covalently associated through relatively weak interactions at the CH3-CH3 interface^24, 45, 46^. As a consequence, monovalent IgG4Δhinge molecules are still able to dimerize in a concentration dependent manner and high target expression levels can promote local dimerization and induce crosslinking. Although this effect is not evident from the results, the 4% blocking observed in 1 animal that only received IgG4Δhinge, compared to untreated animals, could thus be explained. Likewise, local IgG concentrations in the endosomes could promote IgG4Δhinge dimerization and increase the avidity for FcRn. This could explain the discrepancy between the clearance rate of IgG4 half-molecules observed in Balb/c mice in our study and that reported by Wilkinson *et al*.^46^, i.e. a 2.5-fold faster clearance of IgG4Δhinge relative to wild-type IgG4 in this study, compared to a 10–20-fold faster clearance for IgG4 half-molecules engineered to be more monovalent. Although the pharmacokinetic (PK) profile of IgG4Δhinge is superior to that of Fab fragments, functionally monovalent formats with regular IgG PK would still be preferred. A non-glycosylated one-armed format like the one described for c-Met^47^, a non-activating functionally monovalent bispecific IgG format (e.g. containing one dummy arm)^48^, or even a non-activating bispecific IgG containing two non-overlapping AChR-specific arms that supplement each other in blocking auto-antibodies but incapable of inducing AChR-crosslinking, would be worth investigating.

In our study we used a monoclonal antibody to induce disease in our model, while in patients the AChR autoantibodies are polyclonal^49^. The competition between the therapeutic IgG4Δhinge-637 antibody and poly-clonal patient IgG is variable, based on experiments with the scFv-637^9^ and Fab-637^7^. Such an *in vitro* competition assay could be used to identify patients that might benefit from IgG4Δhinge blocker antibodies prior to therapy. Moreover, the blocker therapy could be extended with a number of other human AChR monoclonal antibodies that are now available^7, 49–52^.

With the present study efficacy of the therapy could not be tested in a model of severe clinical disease symptoms since we used non-human primates. Therefore our results do not allow definitive conclusions about the safety and efficacy in severe disease, e.g. a myasthenic crisis. Since the NMJ has a remarkable capacity for regeneration and the AChR has a high turnover of ~1 week, we believe that even during pre-existing disease it is meaningful to prevent antibody-binding to newly-formed AChRs.

Specific conditions in which MG blocker therapy might potentially be beneficial include drug-resistant MG, neonatal myasthenia or arthrogryposis, where passive transfer occurs from mother to child via placental transfer. Furthermore, the IgG4Δhinge format might be useful for treatment of other antibody-driven autoimmune channelopathies^53^, such as neuromyelitis optica^44^, or organ-specific AIDs in general, where complement activation or antigenic modulation are major pathogenic mechanisms.

Taken together, these results demonstrate that IgG4Δhinge shows good therapeutic efficacy in a well-characterized model for PTMG in rhesus monkeys, which is highly supportive of further investigations into the development of antigen-specific therapies.

## Materials and Methods

### Cells

FreeStyle™ 293 F (HEK-293F) and FreeStyle™ CHO-S (CHO-S) cells were cultured in FreeStyle™ 293 expression medium and FreeStyle™ CHO expression medium, respectively (Invitrogen, Carlsbad, CA). TE671 (human rhabdomyosarcoma^54^) cells were cultured in IMDM (Gibco) supplemented with 10% (v/v) heat-inactivated FCS (Bodinco, Alkmaar, the Netherlands), 1% (v/v) penicillin/streptomycin (Gibco), 1 mM pyruvate (Gibco) and 2.5 μM dexamethasone (Sigma).

### Cloning and expression of antibodies

Construction of expression vectors for IgG1-637 (pIgG-637) and IgG4-637 (pTomG4MG and pConLamMG) have been described previously^11^. For the construction of the IgG4Δhinge-637 heavy chain expression vector, the IgG4-637 heavy chain coding sequence was codon optimized with deletion of amino acid residues 216-ESKYGPPCPSCP-230 (EU-numbering conventions are used throughout the manuscript), constituting the genetic IgG4 hinge exon. The whole construct was synthesized de novo by Geneart AG (Regensburg, Germany) and cloned in expression vector pEE6.4 (Lonza Biologics, Slough, UK), resulting in pHG-MG.

All antibodies were produced under serum-free conditions (FreeStyle™ medium) by cotransfecting relevant heavy and light chain expression vectors in HEK-293F cells, using 293fectin (Invitrogen), or CHO-S cells using FreeStyle™ MAX Reagent (Invitrogen), both according to the manufacturer’s instructions. Stable CHO-K1SV clones expressing IgG1-637, IgG4-637 or IgG4Δhinge-637 were obtained after selection with 50 μM MSX.

Antibodies were purified by Protein A affinity chromatography (rProtein A FF, GE Healthcare, Uppsala, Sweden), dialyzed overnight to PBS and filtered-sterilized over 0.2 µM dead-end filters. Concentration of purified IgGs was determined by nephelometry and absorbance at 280 nm. Purified proteins were analyzed by SDS-PAGE, mass spectrometry and glycoanalysis. Batches of IgG were tested by size-exclusion chromatography and shown to be at least 94% monomeric. Endotoxin levels of batches used *in vivo* were below 0.1 EU/mg IgG.

### Antigenic modulation of AChR

Antibody-induced degradation of surface AChR (antigenic modulation) was measured as described^11^. In short, confluent TE671 cells were incubated for 3 h at 37 °C with serial dilutions of IgG1-637, IgG4Δhinge-637, human intravenous immunoglobulin (IVIg; Immunoglubulin I.V., Sanquin, the Netherlands). The antibodies were diluted in DMEM containing 40 μM cycloheximide (blocking de novo AChR synthesis). After washing of the cells, remaining AChR expression was determined by incubating for 1 h at 37 °C with an excess of ^125^I-labeled α-bungarotoxin in the same medium (without antibody), washing three times with PBS and assessing the amount of radioactivity bound. Nonspecific binding was measured by incubating cells with unlabeled α-bungarotoxin prior to incubation with ^125^I-labeled α-bungarotoxin.

### Passive transfer myasthenia gravis in rhesus monkeys

Experiments were approved by the institutional ethical Committee on Animal Welfare (protocol DEC-590 BPRC) of the Biomedical Primate Research Center (Rijswijk, the Netherlands). All animal experimental procedures complied with applicable guidelines, regulations and laws of the Netherlands. Female rhesus monkeys (*Macaca mulatta*) of 3.5 to 8.0 kg were pre-screened for the presence of preexisting anti-human IgG responses and were found negative (data not shown). Antibodies were given as three doses (injected on consecutive days) to guarantee animal safety and enable acute therapy in case of a myasthenic crisis^12^. Intravenous injections with IgG1-637 and IgG4Δhinge-637 were well tolerated and no acute adverse effects were observed. MG was induced by administration of IgG1-637 at doses of 1.7 mg/kg/day resulting in a total cumulative dose of 5 mg/kg. The effect of IgG4Δhinge-637 was tested alone or in combination with IgG1-637 and was administered 6 hours prior to each IgG1-637 injection at 10 mg/kg/day (total cumulative dose of 30 mg/kg). In the case where IgG4Δhinge-637 was tested alone, a dose of saline was administered instead of the IgG1-637. Whole blood samples were taken on different days and collected in clot activator tubes with gel separator (Greiner) and tubes containing a mix of protease inhibitors and anti-coagulation factors^55^. The clot activator tubes were centrifuged at room temperature for 10 min at 2000 × g. The resulting serum was collected and used for clinical chemistry or stored at −80 °C. The tubes containing protease inhibitors and anti-coagulation factors were centrifuged at room temperature for 10 min at 1000 × g, no brake. The resulting plasma was aliquoted into 3 tubes and stored at −80 °C. Each animal was used for an experiment only once to avoid the effect of a primate anti-human antibody response. In total fifteen monkeys (Supplementary Table 1) were used which all survived the experiments and were not sacrificed for analysis.

### Clinical observation

Body weight was recorded each day the animals were sedated. Clinical muscle weakness was defined by at least two of the following symptoms: difficulty to walk, difficulty to climb, difficulty to eat or swallow, drooping of the eyelids (ptosis), more than 5% weight loss. If any one of the aforementioned tasks (walking, climbing or eating/swallowing) could not be performed at all, weakness was also demonstrated. In this study, none of these symptoms were observed.

### Analysis of neuromuscular transmission by single fiber electromyography (SFEMG)

Rhesus monkeys were anesthetized with 10 mg/kg ketamine without the use of muscle relaxants. Stimulated SFEMG was performed in the orbicularis oculi (OO) muscles. As a stimulation electrode, a monopolar needle electrode was placed lateral to the lateral canthus of the eye to stimulate facial nerve branches. Stimulus duration was 0.02 ms and stimulus intensity was increased until a visible movement of the upper eyelid was obtained during 3 Hz stimulation. The recording SF-needle electrode was placed in the orbital part of the OO. As soon as muscle fiber potentials were obtained, stimulation frequency was increased to 10 Hz. All examinations recorded 68–100 sweeps (average was 99.7) of >20 muscle fiber action potentials (MFAPs). Jitter is the measurement of variation of the inter-potential interval. Jitter values were expressed as the mean consecutive difference (MCD), which is defined as the mean time interval between the triggered potential and the time-locked single muscle fiber action potential. Blockings are defined as the absence of a muscle fiber action potential after nerve stimulation. SFEMG was performed at baseline and repeated seven days after administration of antibodies.

### Measurement of decrement of the compound muscle action potential (CMAP)

Decrement of CMAP was measured in the extensor digitorum brevis muscle upon stimulation of the peroneal nerve below the fibular head. Recording and stimulation was performed with surface electrodes. The reference electrode was placed distal to the active recording electrode at a distance of 3 to 4 cm. Using single stimuli of 0.1 ms duration and gradually increasing intensity, the current intensity was determined at which a maximal CMAP amplitude was reached. To detect a decremental response, ten stimuli with a 20 to 30% higher stimulus strength (supramaximal) were given at 3, 5 and 10 Hz. Test results had to be reproducible for at least three consecutive measurements and were considered positive when both the amplitude and the area of the negative peak op the CMAP showed a decrement of at least 10%. Average decrement values of individual animals were also analyzed statistically at the level of experimental groups.

### Intercostal muscle biopsies

Biopsies were taken before and seven days after the first injection of antibody, under general anesthesia (induced with ketamine and maintained with endotracheal isoflurane/halothane/oxygen) and analgesia (Temgesic, 0.3 mg/mL buprenorfine base, Schering Plough B.V.), as originally described for human muscle^56^. Pieces of the external layer of parasternal intercostal muscle were prepared free from the internal layer by means of small non-traumatic forceps through an incision between the sixth and seventh rib. The biopsy was then taken carefully from rib-to-rib with pieces of periosteum attached. Subsequently, intercostal muscle biopsies were cut into fragments of 3 mm diameter. For electron microscopic analysis, biopsies submerged in fixation buffer (0.1 M phosphate buffer supplemented with 2.5% glutaraldehyde, pH 7.4) and stored at 4 °C for up to seven days. For immunofluorescent analysis, biopsies were frozen on melting isopentane and subsequently stored at −80 °C.

### Electron microscopy

Glutaraldehyde-fixed biopsies were then postfixed with 1% osmiumtetroxide (in 0.1 M phosphate buffer, pH 7.4), dehydrated through a graded ethanol series and embedded in epoxy resin (Glycid ether 100, Serva). Endplates were located in toluidine blue-stained semi-thin sections. Ultra-thin sections from selected areas were contrasted with uranyl acetate and lead citrate and analyzed with a Philips CM 100 electron microscope. Quantitative morphometric analysis was performed as previously described^57^ and calculated as the postsynaptic membrane length divided by the presynaptic membrane length (folding index).

Postsynaptic widening of the primary or secondary cleft was scored blindly. The definitions for the ‘cleft widening score’ were: severely widened clefts (primary or secondary) = 0; widened clefts (primary or secondary) = 1, normal or very slightly widened clefts (primary or secondary) = 2, clearly intact postsynaptic membrane = 3. Four to twenty-five (median = ten) neuromuscular junctions were scored per monkey for postsynaptic widening. The percentage of widened postsynaptic clefts was calculated per monkey. In this calculation scores of 0 (severely widened clefts) and 1 (widened clefts) were regarded as proof of postsynaptic cleft widening, while scores of 2 (normal or very slightly widened clefts) and 3 (clearly intact postsynaptic membrane) were considered to have no cleft widening.

### Immunofluorescence staining, microscopy and quantitative analysis

Biopsies were cryosectioned (10 µm) and stored at −80 °C. Sections were fixed with ice-cold acetone for 10 min. The membrane attack complex (MAC) C5b-9 was stained mouse mAb AE11 (1:50, Hycultbiotech, the Netherlands) for one hour. Immunofluorescent triple staining was performed for one hour with FITC-conjugated sheep anti-human IgG (the Binding Site; minimal cross-reaction with monkey immunoglobulins), and Alexa-647-conjugated alpha-bungarotoxin (1:300, Thermo Fischer Scientific, catalog number B35450) and a Alexa Fluor 594 conjugated goat anti-mouse IgG (H + L) secondary antibody (1:500, Thermo Fischer Scientific, catalog number A-11005) which had minimal cross-reaction to other immunoglobulins used in the staining procedure or present in the biopsies. Washes were performed with PBS containing 0.05% Triton-X100 (3 × 5 min). Sections were mounted with 80% glycerol in PBS. Fluorescent photomicrographs of endplate regions were acquired using µManager software 2.0 on an BX51WI spinning disk confocal fluorescence microscope (Olympus, Hamburg, Germany) with an Hamamatsu EM-CCD C9100 digital camera. Fluorescent intensities of endplates were analyzed using imageJ software (www.imagej.nih.gov/ij/) as described^58–60^. All staining procedures and fluorescent analysis were performed on coded samples by a blinded investigator.

### Statistical analysis

Unless otherwise indicated, differences between treatment groups were tested for significance by a linear mixed model using SAS software (SAS Institute Inc., Cary, NC). In this model subject was included as a random effect and group and/or time as fixed effects. Compound symmetry was used as variance/covariance structure. The binary cleft widening data were analyzed by general estimating equations for longitudinal binary outcome data. P-values below 0.05 were considered significant.

## Electronic supplementary material

Losen et al. supplementary material SREP-16-35335A
