# Supplementary material for: Hinge-deleted IgG4 blocker therapy for acetylcholine receptor myasthenia gravis in rhesus monkeys
Source: Sci Rep. 2017 Apr 20;7:992. doi: 10.1038/s41598-017-01019-5 (PMC5430546; doi:10.1038/s41598-017-01019-5)
Supplement: Supplementary file 1 — Losen et al. supplementary material SREP-16-35335A [file 41598_2017_1019_MOESM1_ESM.pdf]

Supplementary Materials for:

Hinge-deleted IgG4 blocker therapy for acetylcholine receptor myasthenia gravis in rhesus monkeys

Mario Losen[1,*,†], Aran F. Labrijn[2,*,†], Vivianne H. van Kranen-Mastenbroek[3,†], Maarten L. Janmaat[2], Krista G. Haanstra[4], Frank J. Beurskens[2], Tom Vink[2], Margreet Jonker[4,5], Bert A. 't Hart[4,6], Marina Mané-Damas[1], Peter C. Molenaar[1], Pilar Martinez-Martinez[1], Eline van der Esch[1], Janine Schuurman[2], Marc H. de Baets[1,7], Paul W.H.I. Parren[2,5]

[1]Department of Psychiatry and Neuropsychology, School for Mental Health and Neuroscience, Maastricht University, Maastricht, the Netherlands; [2]Genmab, Utrecht, the Netherlands; [3]Department of Clinical Neurophysiology, Maastricht University Medical Center; [4]Biomedical Primate Research Centre, Rijswijk, the Netherlands; [5]Department of immunohematology and Blood Transfusion, Leiden University Medical Center, Leiden, the Netherlands; [6]University of Groningen, University Medical Center, Department of Neuroscience, Groningen, the Netherlands; [7]Neuroimmunology Group, Biomedical Research Institute (BIOMED), Hasselt University, Diepenbeek, Belgium.

[†]These authors contributed equally.

*Corresponding authors:

Mario Losen
Department of Psychiatry and Neuropsychology,
Maastricht University, Universiteitssingel 50,
6200 MD Maastricht, the Netherlands
Phone: +31 43 3881039
Fax: +31 43 3884086
e-mail address: m.losen@maastrichtuniversity.nl

Aran F. Labrijn
Genmab,
Yalelaan 60,
3584 CM Utrecht, the Netherlands
Phone: +31 30 2123321
Fax: +31 30 2123110
e-mail address: a.labrijn@genmab.com

Running Title: Hinge-deleted IgG4 blocker therapy in AChR-MG

Cells

Raji (human Burkitt's Lymphoma) cells (ATCC) were cultured in RPMI 1640 (Lonza) supplemented with 10% heat-inactivated cosmic calf serum (CCS; Hyclone), 2 mM L-glutamine (Lonza), 1 mM sodium pyruvate (Lonza), 50 IU/mL penicillin and 50 μg/mL streptomycin (Lonza).

IIA1.6 (mouse pre-B) cells (ATCC) were stably transfected with human FcγRI and human FcRγ-chain (IIA1.6-FcγRI) as described previously (Van Vugt *et al.*, 1998). Cells were cultured in RPMI 1640 supplemented with 10% heat-inactivated fetal calf serum (FCS; Bodinco), 50 IU/mL penicillin, 50 μg/mL streptomycin (Lonza) and 2.5 μg/mL methotrexate (Teva Pharmachemie).

Cloning and expression of antibodies

Construction of expression vectors for IgG1-CD20, IgG4-CD20, IgG1-EGFR, IgG4-EGFR and IgG4Δhinge-EGFR have been described previously (van der Neut Kolfschoten *et al.*, 2007; Labrijn *et al.*, 2009; Rose *et al.*, 2011). For the construction of the IgG4Δhinge-CD20 heavy chain expression vector, IgG4-CD20 heavy chain coding sequence was codon-optimized with deletion of amino acid residues 216-ESKYGPPCPSCP-230 (EU-numbering conventions are used throughout the manuscript), constituting the genetic IgG4 hinge exon. The whole construct was synthesized de novo by Geneart AG (Regensburg, Germany) and cloned in expression vector pEE6.4 (Lonza Biologics, Slough, UK), resulting in pHG-7D8, respectively.

Complement binding and complement-dependent cytotoxicity (CDC)

The capacity of C1q to bind to plate-bound mAbs was assessed by ELISA as described previously (25). In short, ELISA plates (Greiner bio-one) were coated overnight with serial

diluted mAbs (0.06 µg/mL - 10 µg/mL) in PBS at 4 °C. After blocking, purified C1q (Sigma) was added at 2 µg/ml and incubated for 1 h at 37 °C. The plates were washed and bound C1q was detected by incubating (1 h at 21 °C) with rabbit anti-human C1q polyclonal antibodies (DAKO), followed by subsequent incubations with HRP-labeled swine-anti-rabbit IgG Fc (DAKO; 1 h 21 °C) and ABTS substrate (Roche Diagnostics). The color development reaction was stopped (after 15-30 min) by addition of an equal volume of oxalic acid (Riedel de Haen) and absorbance was measured at 405 nm. Non-linear regression curve-fitting was used to calculate binding curves (GraphPad Software).

The capacity to induce CDC was assessed essentially as described (25). Briefly, target cells ($1x10^5$ Raji cells) were pre-incubated at 21 °C for 15 min with 133.3 nM antibodies. Pooled human serum (20%) was added as a source of complement and cells were incubated at 37 °C for an additional 45 min. Cells were then put on ice and viability was determined by staining with propidium iodide (PI) and detected using a FACSCanto II flow cytometer (BD Biosciences).

FcyRI binding

IIA1.6 cells stably transfected with FcγRI were incubated with for 30 min at 4 °C with serial diluted antibodies. Cells were washed with FACS buffer and stained for 30 minutes at 4 °C with PE-labelled goat anti-human IgG F(ab')2 fragments. Unbound antibodies were removed by washing. Mean fluorescence (PE positivity) of the cells was measured using a FACS Canto II flow cytometer.

Pharmacokinetic studies in mice

Balb/c mice were obtained from Charles River Laboratories (Maastricht, the Netherlands) and mFcRn-/- hFcRn+ mice, deficient in murine (m)FcRn and carrying a human (h)FcRn

transgene (Tg) [B6.Cg-Fcgrt^{tm1Dcr} Tg(FCGRT)276Dcr], were described elsewhere (Petkova *et al.*, 2006) and obtained from Jackson Laboratory (Bar Harbor, ME). The mice (6-8 weeks old females) were housed in a barrier unit of the Central Laboratory Animal Facility (Utrecht, the Netherlands) and kept in filter-top cages with water and food provided ad libitum. All experiments were approved by the Utrecht University animal ethics committee.

Antibodies (5 mg/kg) were administered via a single, intravenous tail vein injection and blood samples were drawn from the saphenal vein at different time-point after administration. Blood was collected in heparin-containing vials, which were kept on ice, and centrifuged (5 minutes at 10,000 x g) to separate the plasma from cells. Plasma was transferred to a new vial and stored at -20 °C for determination of antibody levels.

Cynomolgus monkeys

Experiments in cynomolgus monkeys (*Macaca fascicularis*) were conducted at the Charles River Laboratories (Tranent, UK) in accordance with the European Convention for the Protection of Vertebrate Animals Used for Experimental and Other Scientific Purposes (Council of Europe), under control of the UK Home Office. Two male and 2 female weighing 2.4 to 2.7 kg were pre-screened for the presence of preexisting anti-human IgG responses and were found negative (data not shown).

Pharmacokinetic studies in cynomolgus monkeys

Antibodies (20 mg/kg) were administered by slow intravenous tail vein injection (10 mL/kg body weight/ 15 minutes). Blood samples (~ 1 mL) were collected from the femoral vein at different time-point after administration using sterile, disposable plastic syringes and hypodermic needles. After collection, the samples were transferred to glass tubes without

anticoagulant and allowed to clot for at least 1 h. The samples were then centrifuged (10 minutes 1500 x g at 4 °C) to separate the serum from cells. Serum was transferred to a new tube and stored at -80 °C for determination of antibody levels.

Quantitative IgG ELISA

The total amount of human antibodies in the mice was determined by sandwich ELISA. In short, ELISA plates were coated overnight with 2 µg/mL of mouse anti-human IgG (MH16-1; Sanquin) in PBS at 4 °C. The plates were subsequently washed and blocked with PBS-C (PBS/ 2% normal chicken serum; Gibco) for 1 h at 37 °C. Next, the plates were washed and incubated with diluted plasma samples in PBS-TC (PBS/ 0.05% Tween-20/ 2% normal chicken serum) for 120 min at 20 °C under shaking conditions (300 rpm). Bound antibodies were detected by HRP-labeled goat anti-human IgG (Jackson ImmunoResearch) and ABTS substrate (Roche Diagnostics). The color development reaction was stopped by addition of an equal volume of oxalic acid and absorbance was measured at 405 nm. IgG was quantified by nonlinear regression curve-fitting (GraphPad PRISM v.5.01; GraphPad Software) using the injection mixtures as reference.

Serum concentrations of human antibodies in the cynomolgus monkeys were determined by sandwich ELISA. In brief, plates were coated overnight with 1 µg/mL FITC-labelled monkey pre-absorbed swine anti-human IgG (The Binding Site) at 4 °C. The plates were subsequently washed and blocked with PBS-C for 1 h at 37 °C. Next, the plates were washed and incubated with diluted serum samples (1:50, 1:150, 1:450) in PBS-TC for 60 min at 20 °C under shaking conditions (300 rpm). Bound antibodies were detected by incubation with biotin-labeled anti-human IgG4 (BD Biosciences; diluted 1:20,000 in PBS-TC), followed by HRP-conjugated Streptavidin (Sanquin; 1:10,000 in PBS-TC) and ABTS substrate. The color development

reaction was stopped by addition of an equal volume of oxalic acid and absorbance was measured at 405 nm. IgG was quantified by nonlinear regression curve-fitting (GraphPad PRISM v.5.01; GraphPad Software) using a 2-fold serial dilution series of IgG4-637 and IgG4Δhinge as reference curve.

Pharmacokinetic data analysis

Plasma clearance was calculated for individual animals as Dose (in μg) / AUC(0-∞) per kg body weight (ml/day/kg). For this, the area under the curve (AUC) from time zero to time 't' (AUC(0-t)), was determined from the plasma antibody concentration versus time plot. Additionally, the Kel (terminal elimination rate constants) were determined from the log-concentration over time curves using the terminal parts (> 2 days) of the curves by nonlinear regression curve-fitting (GraphPad PRISM v.5.01; GraphPad Software). AUC(0-∞) was calculated with the formula AUC(0-∞) = AUC(0-t) + [Ct / Kel] where Ct is serum concentration at time 't'.

C4b/c Enzyme-linked immunosorbent assay (ELISA)

Overall complement activation was measured by a quantitative ELISA assessing the amount of C4b/bi/c (abbreviated further as C4b/c) (Wolbink *et al.*, 1993). Briefly, mAb anti-C4-1 (Sanquin), recognizing a neoepitope on activated C4, was coated overnight and used as capture antibody. Several dilutions of rhesus plasma sample (1/10, 1/30, and 1/90) were subsequently incubated for 1 hour at 4 °C. Biotinylated polyclonal sheep anti-human C4b/c antibody (Sanquin) in combination with streptavidin-HRP (GE Healthcare) was used for detection. The ELISA was developed using TMB substrate (Merck) and stopped by addition of 2M H_2SO_4. After measuring absorbance at 450 nm, the amount of C4b/c was quantified by non-linear regression curve fitting (GraphPad PRISM v.5.01; GraphPad Software) using aged human serum

(Sanquin), which contains a known amount of activated C4 (stock concentration 25 AU/mL), as reference curve.

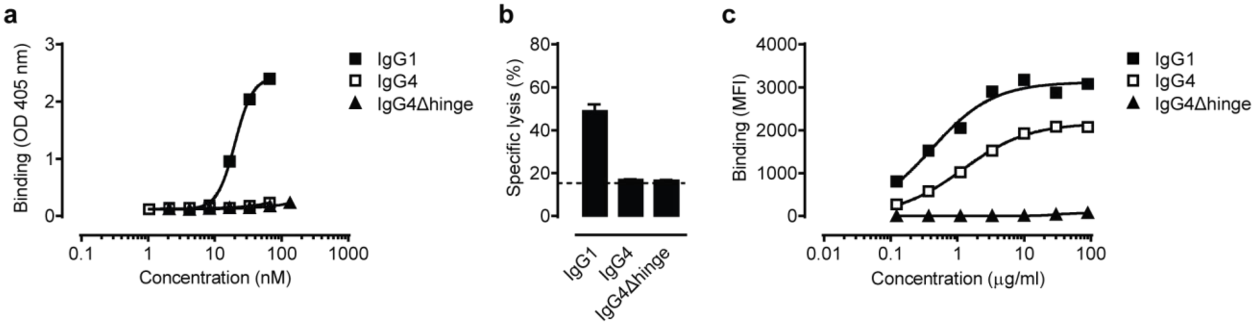

Supplementary Figure 1 Functional characterization of IgG4Δhinge. (a) Binding of serial diluted C1q to immobilized antibody variants of mAb 7D8 as determined by ELISA. (b) CDC of Raji cells incubated for 45 min at 37 °C with antibody variants of mAb 7D8 at 10 µg/mL in the presence of 20% pooled human serum. The dashed line indicates background lysis in the absence of antibodies. (c) Binding of antibody variants of mAb 7D8 to FcγRI stably expressed on IIA1.6 cells as determined by flow-cytometry. One representative experiment out of two is shown.

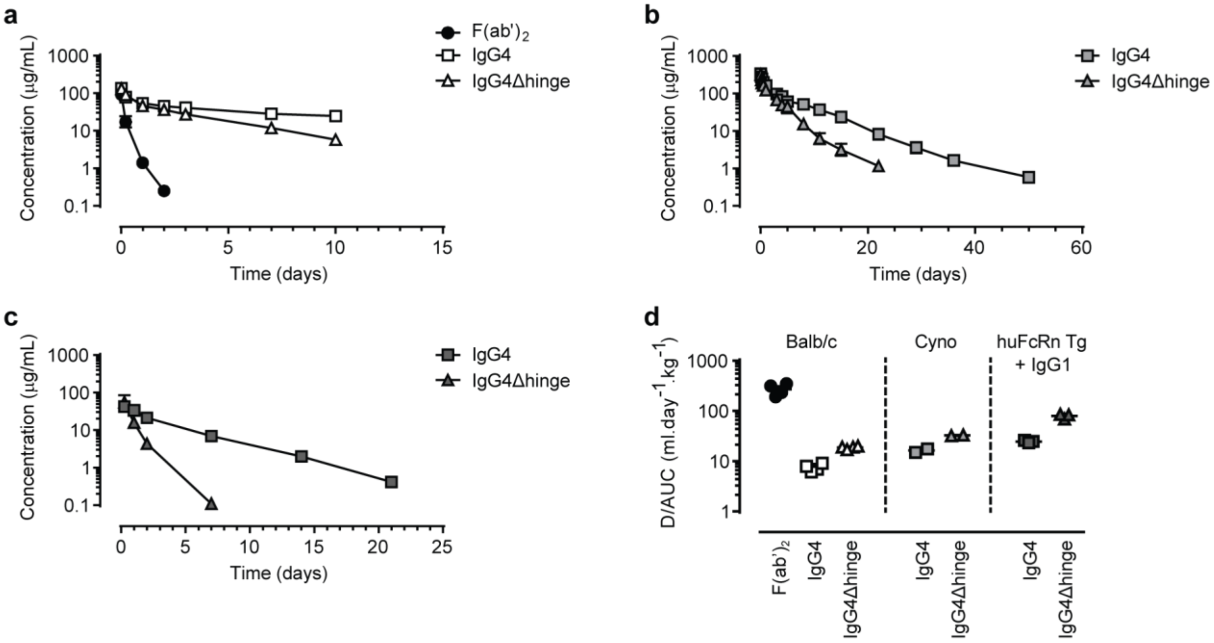

Supplementary Figure 2 Pharmacokinetics of IgG4Δhinge in different species. Plasma concentrations of single injections of IgG4-637 or IgG4Δhinge-637 in (a) Balb/c mice (5 mg/kg dose), (b) cynomolgus monkeys (20 mg/kg dose) or (c) human FcRn transgenic (Tg) mice (5 mg/kg dose) were monitored over time by ELISA. F(ab’)$_2$ fragments of IgG1-637 were included as reference in Balb/c mice. (d) Plasma clearance rates were calculated in different species. D = dose; AUC = area under curve.

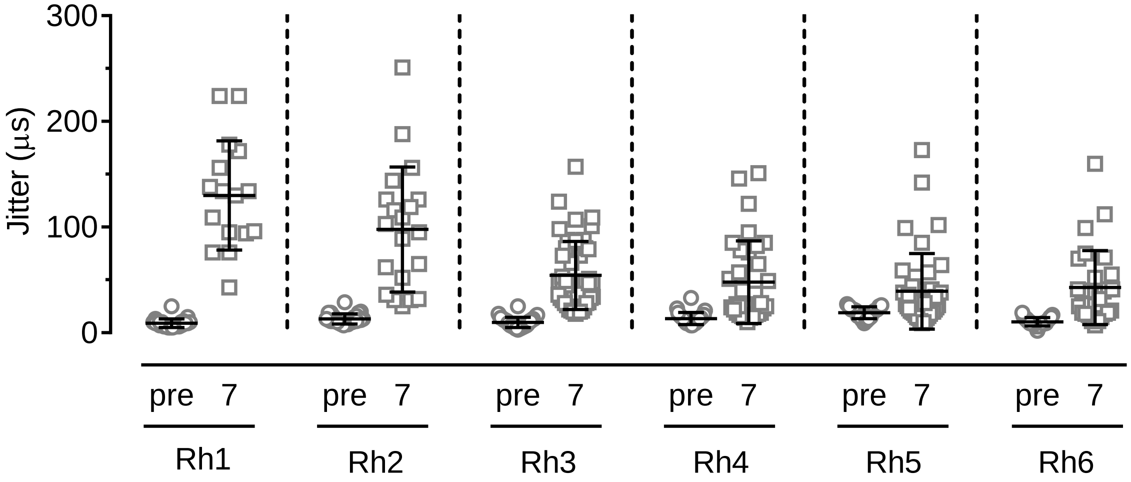

Supplementary Figure 3 Single-fiber electromyography (SFEMG) of rhesus monkeys. SFEMG jitter measurements of orbicularis oculi muscles of 6 rhesus monkeys (Rh1-Rh6, see Supplementary Table 1) that were injected with a cumulative dose of 5 mg/kg IgG1-637 per animal. The jitter measurements were performed before injections ('pre') and again seven days after the first injection of IgG1-637 ('7'). Jitter values were expressed as the mean consecutive difference, which is defined as the mean time-interval between the triggered potential and the time-locked second single muscle fiber action potential.

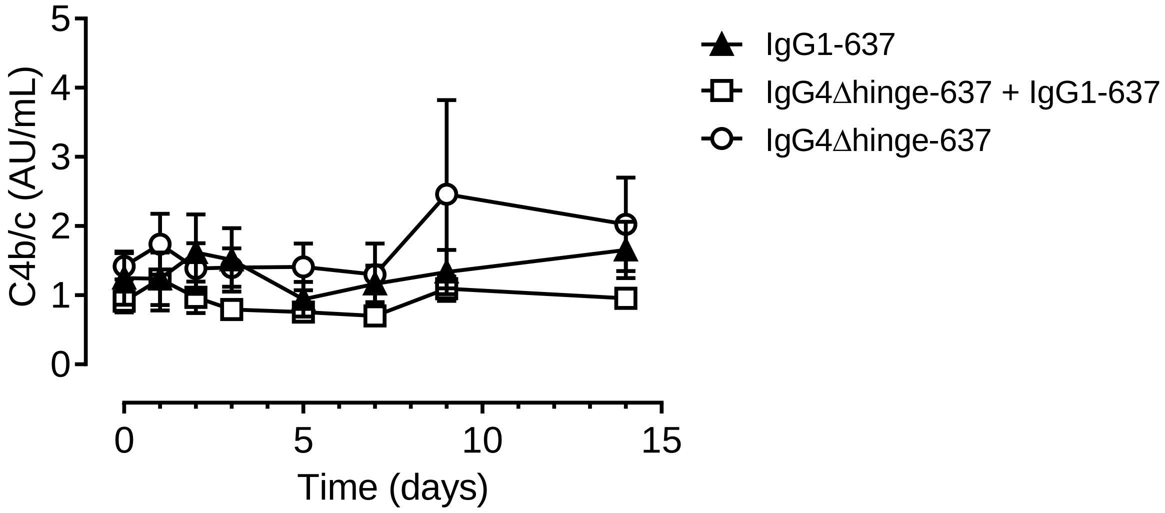

Supplementary Fig. 4. C4b/c levels in rhesus monkey plasma. The levels of activated complement factor C4b/c were measured by ELISA in the plasma of all animals at different time-point. Levels are expressed as AU/mL. Data-points represent mean ± SEM.

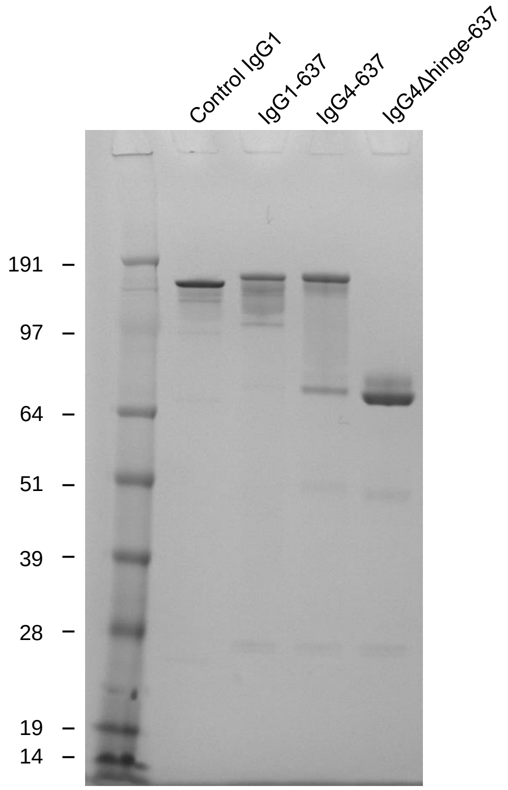

Supplementary Figure 5. Full length version of Figure 1B displaying purified human IgG1, IgG4 and IgG4Δhinge variants of mAb 637 analyzed by non-reducing SDS-PAGE (4-12% Bis-Tris Gel). A control IgG1 antibody was included for reference.

Table S1. Animal identification and demographics.

No.	Group[a]	Set[b]	Animal ID	Birth date (mm-dd-yyyy)	Weight at day 0 (gram)	Sex	Origin
Rh1	PS	1	Ri201004	01-01-2002	5550	Female	Import, China
Rh2	PS	1	Ri204102	05-04-2002	5550	Female	Import, China
Rh3	PS	1	Ri212242	11-12-2002	7850	Female	Import, unknown
Rh4	PS	2	Ri201108	06-01-2002	5000	Female	Import, China
Rh5	PS	2	Ri202224	10-02-2002	5100	Female	Import, China
Rh6	PS	2	Ri202228	10-02-2002	6600	Female	Import, China
Rh7	TS-A1	5	Ri0511022	01-11-2005	4550	Female	Import, China
Rh8	TS-A1	3	Ri0511130	04-11-2005	4550	Female	Import, China
Rh9	TS-A1	4	Ri0511134	05-11-2005	4650	Female	Import, China
Rh10	TS-A1	3	Ri0512110	04-12-2005	3750	Female	Import, China
Rh11	TS-A1	4	Ri0512118	04-12-2005	3850	Female	Import, China
Rh12	TS-A1	5	Ri211008	01-11-2002	8050	Female	Import, China
Rh13	TS-A2	4	Ri0507014	01-07-2005	5750	Female	Import, China
Rh14	TS-A2	3	Ri0511008	01-11-2005	4400	Female	Import, China
Rh15	TS-A3	5	Ri0511002	01-11-2005	3650	Female	Import, China

[a]PS = pilot-study (IgG1), TS = treatment study, A1 = IgG1 + IgG4Δhinge, A2 = IgG4Δhinge + PBS , A3 = PBS + IgG1
[b]Animals were treated in sets (numbered sequentially 1-5) of three or four at the same time.

Table S2. Electrophysiology results of CMAP and SFEMG

Animal number	Prescreening				t=7 days			
	amplitude decrement	area decrement	jitter [µs]	blocking [%]	amplitude decrement	area decrement	jitter [µs]	blocking [%]
Rh1	n.a.	n.a.	9	0	**9.6**	**13.8**	**162**	**70.3**
Rh2	n.a.	n.a.	11	0	**22.8**	**25**	**89**	**32.1**
Rh3	n.a.	n.a.	11	0	**32.7**	**33.7**	**59**	**11.5**
Rh4	n.a.	n.a.	14	0	**8.5**	**7.5**	**50**	**5.8**
Rh5	n.a.	n.a.	19	0	2.0	3.7	**40**	**4.5**
Rh6	n.a.	n.a.	10	0	2.0	6.0	**28**	0.0
Rh7	1.0	2.3	15	0	-1.0	1.3	**35**	**12.3**
Rh8	0.0	2.3	16	0	-1.3	0.7	**23**	0.0
Rh9	-0.3	1.3	16	0	0.0	0.7	**33**	**4.7**
Rh10	-1.3	1.7	16	0	-1.7	0.3	**62**	**15.3**
Rh11	0.0	1.0	16	0	0.0	1.0	**20**	**0.4**
Rh12	0.0	1.7	14	0	1.8	4.3	**46**	**18.7**
Rh13	-0.5	3.5	14	0	1.7	3.4	14	0.0
Rh14	-2.7	2.0	19	0	0.7	1.3	**29**	**4.0**
Rh15	0.7	2.0	17	0	-0.7	1.3	**99**	**23.5**

Amplitude and area decrement results of the compound muscle action potentials were measured during 3 Hz nerve stimulation. SFEMG recordings were performed during 10 Hz nerve stimulations. Abnormal values are indicated in bold. n.a.: values not available. All values were obtained from blinded re-analysis of measurements.
